# Supplementary material for: Transcriptomic and Co-Expression Network Profiling of Shoot Apical Meristem Reveal Contrasting Response to Nitrogen Rate between Indica and Japonica Rice Subspecies
Source: Int J Mol Sci. 2019 Nov 25;20(23):5922. doi: 10.3390/ijms20235922 (PMC6928681; doi:10.3390/ijms20235922)
Supplement: Supplementary file 1 [file ijms-20-05922-s001.zip › Figure S1-12 + Table S1-15/Figure S1.pdf]

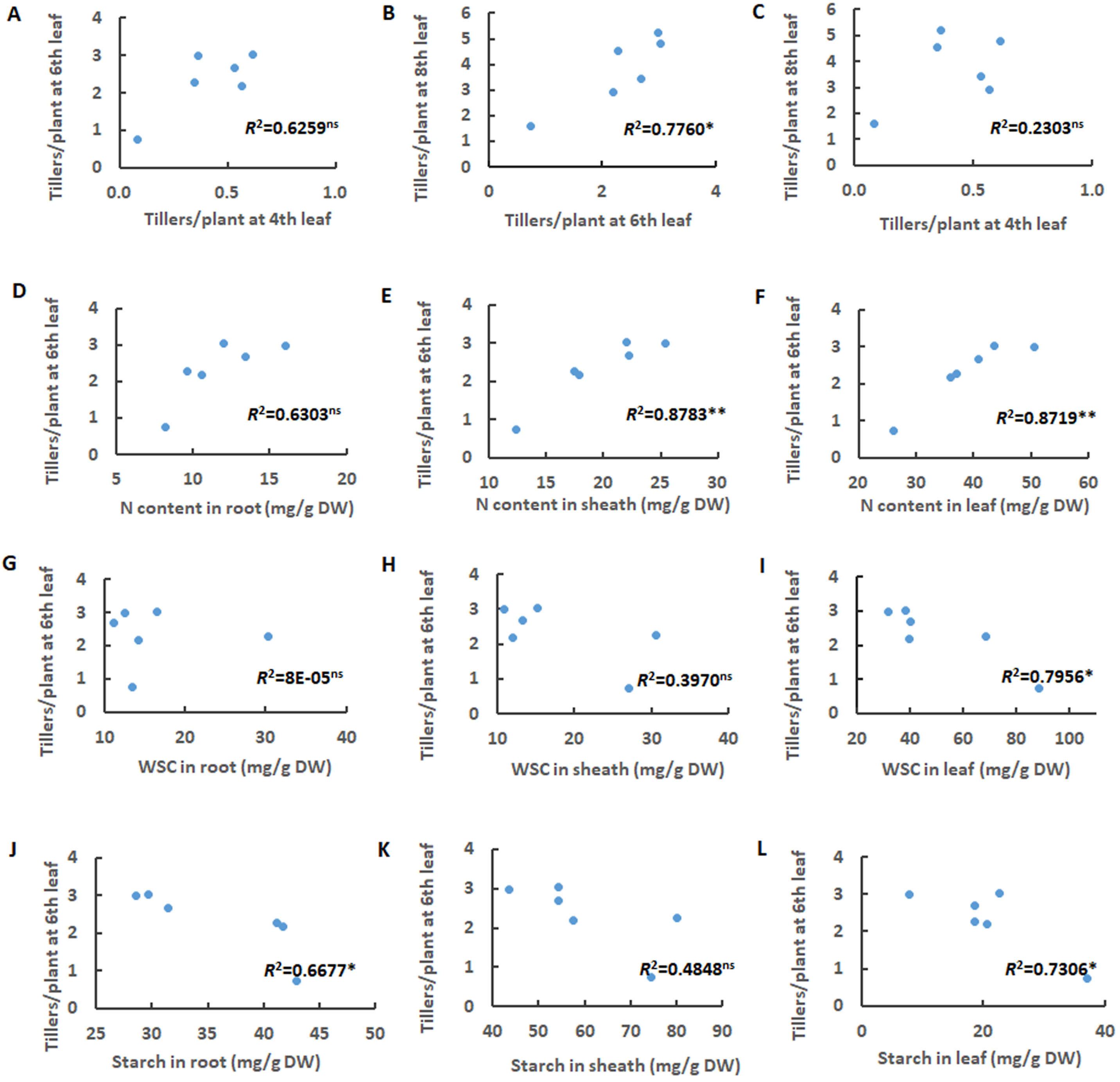

Figure S1. Correlation between tiller numbers and N content, SWC, starch and other traits. (A-C) Correlation between tillers at different leaf ages; (D-F) Correlation between tillering and N content in root, sheaf and leaf organs; (G-I) Correlation between tillering and WSC in root, sheaf and leaf organs; (J-L) Correlation between tillering and starch content in root, sheaf and leaf organs.
