# Supplementary material for: Transcriptomic and Co-Expression Network Profiling of Shoot Apical Meristem Reveal Contrasting Response to Nitrogen Rate between Indica and Japonica Rice Subspecies
Source: Int J Mol Sci. 2019 Nov 25;20(23):5922. doi: 10.3390/ijms20235922 (PMC6928681; doi:10.3390/ijms20235922)
Supplement: Supplementary file 1 [file ijms-20-05922-s001.zip › Figure S1-12 + Table S1-15/Figure S10.pdf]

Figure S10. Expression level (FPKM) of NAM family genes and their differences between the varieties and in response to N rate

Locus	NPB			YD6			P value		
LOC_#	LN	MN	HN	LN	MN	HN	Variety	N rate	Variety x N
Os01g23710	1.1	0.9	1.1	0.5	0.4	0.3	0.005	0.620	0.780
Os01g48446	12.9	8.4	13.1	13.4	7.6	7.6	0.152	0.032	0.172
Os01g64310	1.9	2.8	1.3	0.4	0.5	1.1	0.024	0.652	0.250
Os01g66490	14.9	13.6	15.1	19.8	15.9	11.3	0.201	0.016	0.012
Os01g70110	49.2	43.4	48.7	100.5	90.2	80.5	0.000	0.059	0.073
Os02g15340	1.7	2.3	2.0	1.6	1.4	0.7	0.011	0.262	0.157
Os02g34970	1.5	3.3	3.0	1.6	2.8	3.9	0.467	0.000	0.047
Os02g38130	7.8	7.9	6.8	5.1	5.7	6.9	0.006	0.686	0.061
Os02g41450	2.2	2.9	3.6	1.3	1.4	1.1	0.000	0.074	0.028
Os03g21030	34.8	36.5	33.7	26.0	19.3	27.5	0.000	0.313	0.050
Os04g35660	0.7	1.5	1.5	2.2	4.2	2.7	0.000	0.001	0.022
Os05g34600	4.7	4.4	4.6	8.2	5.6	2.5	0.187	0.021	0.024
Os06g01230	3.3	2.1	2.0	2.8	2.1	1.3	0.144	0.009	0.524
Os06g36480	1.7	1.7	1.8	2.1	2.1	2.1	0.003	0.852	0.869
Os07g48550	2.0	2.1	1.7	1.0	1.0	1.2	0.001	0.943	0.355
Os08g06140	97.2	72.3	64.7	83.3	58.5	61.3	0.129	0.017	0.718
Os08g42400	16.4	16.9	19.4	22.1	25.9	22.5	0.003	0.390	0.230
Os09g33490	8.4	6.9	8.1	12.4	11.8	8.5	0.008	0.173	0.126
Os09g38010	2.5	2.2	2.0	5.1	3.1	2.7	0.001	0.005	0.023
Os10g21560	16.9	14.8	10.6	7.4	6.1	8.7	0.004	0.404	0.143
Os10g27360	0.1	0.2	0.1	0.6	1.2	1.5	0.000	0.065	0.080
Os10g33760	7.0	3.3	8.5	2.7	4.7	6.5	0.108	0.036	0.090
Os10g42130	2.3	1.8	2.0	4.2	3.2	1.9	0.001	0.003	0.010
Os12g05990	0.3	1.0	1.0	0.1	0.4	0.7	0.042	0.022	0.470
Os12g29330	37.0	59.2	50.5	26.6	26.2	50.2	0.002	0.005	0.009
