# Supplementary material for: Transcriptomic and Co-Expression Network Profiling of Shoot Apical Meristem Reveal Contrasting Response to Nitrogen Rate between Indica and Japonica Rice Subspecies
Source: Int J Mol Sci. 2019 Nov 25;20(23):5922. doi: 10.3390/ijms20235922 (PMC6928681; doi:10.3390/ijms20235922)
Supplement: Supplementary file 1 [file ijms-20-05922-s001.zip › Figure S1-12 + Table S1-15/Figure S11.pdf]

Figure S11. Selected carbohydate metabolism related gene expression changes between variety and in response to N rate.

	LOC_#	NPB			YD6			*P* value		
		LN	MN	HN	LN	MN	HN	Variety	N rate	Variety x N
Starch	Os01g52250	8.4	8.2	8.9	5.6	6.5	6.5	0.000	0.267	0.414
	Os02g32660	5.9	4.3	4.0	1.2	1.1	0.9	0.000	0.005	0.012
	Os02g51070	23.9	25.5	25.3	12.5	16.6	22.8	0.002	0.043	0.106
	Os03g55090	121.7	85.3	87.0	91.5	74.8	41.5	0.000	0.000	0.001
	Os04g53310	18.5	20.3	26.7	13.2	19.5	24.9	0.316	0.041	0.734
	Os05g03040	26.8	25.9	23.3	45.4	54.8	35.3	0.002	0.114	0.241
	Os05g37450	12.5	15.1	13.8	6.9	10.0	19.1	0.330	0.050	0.072
	Os06g03990	13.2	13.4	16.5	14.1	15.1	19.4	0.068	0.011	0.620
	Os06g06560	12.8	13.2	15.6	11.6	13.3	14.0	0.162	0.027	0.500
	Os07g22930	67.0	72.7	95.6	45.5	56.2	86.5	0.106	0.032	0.831
	Os08g09230	11.0	8.7	9.3	13.2	14.2	10.6	0.008	0.137	0.137
	Os10g30156	17.1	17.8	20.9	17.0	19.8	26.4	0.039	0.003	0.120
Fructose	Os01g02880	96.2	101.6	108.3	89.1	102.7	102.9	0.245	0.027	0.524
	Os01g67860	653.1	684.3	841.3	618.9	725.5	826.5	0.944	0.011	0.686
	Os02g48360	83.9	91.9	105.8	68.8	83.8	92.8	0.021	0.009	0.761
	Os05g33380	592.6	661.9	769.1	619.2	729.0	745.0	0.248	0.001	0.202
	Os06g45370	4.2	4.7	4.4	5.7	5.8	6.8	0.000	0.107	0.121
	Os08g25720	111.9	93.4	108.6	149.4	142.5	135.7	0.008	0.584	0.669
	Os09g12650	0.4	0.4	0.2	0.2	0.2	0.1	0.026	0.120	0.991
	Os10g08022	0.2	0.5	0.3	0.6	0.8	0.7	0.004	0.204	0.983
	Os11g03900	18.6	16.4	15.5	12.5	11.7	12.3	0.001	0.277	0.410
	Os12g03720	22.7	19.9	19.1	14.4	14.2	14.3	0.000	0.248	0.285
Ribose and Ribulose	Os01g58020	0.4	1.2	1.0	2.9	2.1	2.8	0.031	0.955	0.574
	Os03g07300	8.5	9.2	10.9	7.7	9.6	12.9	0.558	0.030	0.459
	Os03g30300	3.0	3.2	3.3	1.6	1.7	2.3	0.002	0.339	0.628
	Os04g19740	10.7	10.9	10.4	4.0	4.5	3.4	0.000	0.172	0.737
	Os04g57400	32.0	49.9	51.5	26.1	42.6	48.8	0.130	0.003	0.812
	Os04g57410	5.1	9.0	7.9	5.5	7.6	10.1	0.561	0.006	0.146
	Os05g35330	0.0	0.0	0.1	1.0	1.2	0.8	0.007	0.929	0.749
	Os06g04270	112.2	115.7	128.2	106.7	129.4	126.5	0.632	0.036	0.238
	Os07g08030	35.1	42.3	48.9	34.2	38.6	41.2	0.145	0.038	0.562
	Os07g09190	14.2	42.5	25.4	4.5	12.4	47.6	0.256	0.008	0.011
	Os07g41280	15.1	14.5	15.8	30.0	26.4	15.2	0.022	0.198	0.139
	Os08g05830	60.7	64.5	69.8	51.6	56.4	58.1	0.000	0.005	0.473
	Os08g43370	11.2	12.4	16.7	9.6	10.9	14.9	0.072	0.003	0.981
	Os09g32810	50.7	59.3	57.9	62.8	67.8	62.0	0.031	0.249	0.575
	Os11g29400	59.1	75.7	85.7	49.3	65.5	76.7	0.022	0.001	0.988
	Os12g10580	0.1	0.2	0.1	0.3	0.3	0.4	0.023	0.783	0.470
Glucose	Os01g15910	0.3	0.5	0.3	0.5	0.6	0.5	0.005	0.036	0.777
	Os01g26920	7.9	7.9	9.3	6.4	7.5	7.9	0.036	0.069	0.530
	Os01g44220	6.5	5.2	4.4	5.7	6.0	4.7	0.792	0.036	0.286
	Os02g02560	3.0	3.1	2.7	3.5	4.9	2.8	0.028	0.037	0.111
	Os02g38840	15.5	16.6	16.8	8.1	12.2	6.7	0.005	0.409	0.459
	Os02g44510	60.1	55.6	53.5	63.0	69.2	60.9	0.044	0.405	0.429
	Os03g20300	11.5	11.5	13.5	9.3	10.6	11.7	0.030	0.049	0.649
	Os03g29950	2.2	1.8	2.0	1.3	1.5	1.2	0.015	0.849	0.456
	Os03g56460	59.9	55.4	59.8	48.0	51.1	46.3	0.023	0.972	0.510
	Os04g40874	53.0	46.5	52.1	36.1	36.7	27.0	0.002	0.510	0.251
	Os04g49250	1.7	1.7	1.8	0.9	0.9	0.7	0.001	0.980	0.629
	Os04g52370	35.1	31.8	31.1	28.8	29.2	25.3	0.002	0.052	0.310

Glucose	Os05g42070	1.6	2.1	2.6	0.9	1.3	1.8	0.050	0.108	0.997
	Os05g50380	93.9	87.9	111.8	52.3	76.6	89.5	0.013	0.051	0.291
	Os06g10880	6.8	8.3	8.1	5.9	5.2	5.0	0.003	0.763	0.174
	Os06g39260	36.8	44.7	59.0	38.0	51.9	61.4	0.463	0.018	0.851
	Os06g50300	148.3	173.4	227.2	110.8	161.6	188.4	0.053	0.006	0.621
	Os07g13980	6.8	7.0	8.3	6.1	7.3	8.9	0.861	0.028	0.571
	Os07g22350	30.6	32.1	36.5	21.5	26.3	26.6	0.001	0.029	0.418
	Os08g25734	59.8	69.8	80.1	51.9	75.3	108.3	0.234	0.009	0.151
	Os08g37380	3.3	2.9	2.5	3.8	4.5	3.4	0.000	0.001	0.009
	Os09g12660	115.0	100.2	111.6	80.2	83.3	82.6	0.002	0.610	0.404
	Os09g27900	15.2	15.7	17.7	25.3	20.5	16.4	0.021	0.262	0.053
	Os12g25690	90.5	90.2	97.7	63.7	71.7	66.7	0.019	0.868	0.815
Glycerol	Os01g13570	1.0	1.3	1.3	0.7	0.8	0.7	0.005	0.380	0.473
	Os01g19390	1.3	1.1	1.2	1.5	1.6	1.1	0.017	0.048	0.034
	Os01g44069	5.1	5.7	6.2	1.7	2.3	3.2	0.000	0.131	0.883
	Os01g47190	3.9	3.1	1.8	4.0	2.6	0.9	0.235	0.003	0.506
	Os01g57350	1.0	0.8	0.6	1.0	0.9	0.4	0.389	0.000	0.071
	Os01g58610	0.4	0.6	0.6	1.0	1.0	0.6	0.012	0.321	0.095
	Os01g58740	23.8	23.8	24.0	22.7	25.1	27.0	0.089	0.036	0.059
	Os01g63580	20.4	22.5	26.7	12.5	15.8	24.2	0.001	0.000	0.089
	Os01g71280	13.7	13.1	14.5	8.4	9.3	10.9	0.001	0.162	0.561
	Os02g02340	3.7	3.5	2.9	6.7	3.7	3.4	0.005	0.003	0.018
	Os02g07260	310.2	372.6	449.0	284.7	360.5	392.7	0.245	0.017	0.758
	Os02g31030	5.5	6.1	8.7	7.9	5.7	12.5	0.151	0.032	0.365
	Os02g37590	79.6	73.0	75.5	58.4	59.4	67.7	0.004	0.414	0.287
	Os02g43700	272.0	431.6	331.3	108.4	156.8	285.8	0.033	0.271	0.342
	Os02g51590	13.8	14.5	14.8	18.4	19.5	19.4	0.000	0.239	0.898
	Os03g01180	3.6	5.0	5.1	3.2	3.7	4.5	0.020	0.008	0.404
	Os03g03400	22.0	22.5	24.1	15.7	17.2	17.9	0.000	0.160	0.837
	Os03g03720	76.0	69.9	56.2	32.4	35.8	34.8	0.006	0.653	0.556
	Os03g11560	14.8	9.4	8.3	10.9	7.4	5.7	0.003	0.000	0.445
	Os03g15120	37.1	38.7	43.2	42.7	47.1	48.0	0.000	0.004	0.241
	Os03g16140	34.4	33.2	31.0	33.2	33.3	36.4	0.131	0.843	0.033
	Os03g22670	0.2	0.2	0.2	0.0	0.1	0.1	0.000	0.536	0.153
	Os03g31180	5.0	3.3	2.9	5.7	3.4	1.9	0.840	0.000	0.030
	Os03g40670	2.6	3.5	3.6	5.8	4.5	3.9	0.001	0.380	0.007
	Os03g54230	0.3	0.4	0.8	1.9	2.5	1.9	0.011	0.805	0.749
	Os03g58260	12.8	14.0	14.6	11.6	11.4	13.5	0.000	0.001	0.032
	Os03g58290	10.9	12.2	4.8	3.1	2.3	4.7	0.013	0.460	0.120
	Os03g58300	9.3	15.1	9.8	3.7	7.4	10.1	0.006	0.023	0.045
	Os04g01230	45.3	43.9	47.5	34.2	41.9	38.2	0.000	0.004	0.001
	Os04g14760	24.8	26.1	26.2	30.0	30.7	30.8	0.022	0.834	0.987
	Os04g34000	4.5	3.9	4.7	4.8	5.0	5.3	0.006	0.063	0.287
	Os04g39270	20.6	17.7	18.3	24.7	20.2	18.4	0.037	0.012	0.222
	Os04g39610	34.8	32.6	33.0	26.6	23.1	24.0	0.002	0.429	0.959
	Os04g40950	1353	1568	1908	1271	1585	1811	0.507	0.003	0.811
	Os04g52710	15.3	17.0	17.9	15.1	17.3	18.2	0.728	0.002	0.790
	Os04g54200	8.6	8.3	7.1	41.5	40.2	45.3	0.000	0.783	0.506
	Os04g55720	93.7	93.9	112.8	95.4	100.1	108.9	0.765	0.038	0.640
	Os05g04960	72.1	73.8	61.3	56.4	35.2	46.4	0.036	0.567	0.483
	Os05g40420	251.0	260.7	302.8	247.0	268.1	283.5	0.591	0.022	0.537
	Os06g01950	0.0	0.2	0.1	0.2	0.3	0.3	0.042	0.189	0.784
	Os06g29650	1.8	1.6	1.6	0.0	0.2	0.1	0.000	0.922	0.418
	Os06g44460	7.8	7.2	8.3	4.5	4.5	4.8	0.000	0.301	0.614
	Os06g45590	4.8	4.2	4.4	3.2	3.3	2.4	0.000	0.071	0.061
	Os07g08430	35.3	40.2	46.4	33.7	38.8	47.0	0.511	0.000	0.676

	Os07g37580	11.3	11.3	9.0	9.7	9.5	8.9	0.037	0.045	0.250
	Os08g04800	4.2	17.9	9.5	0.7	1.1	8.2	0.001	0.007	0.005
Glycerol	Os08g08110	7.8	7.2	8.8	12.1	11.1	13.8	0.000	0.075	0.821
	Os08g15090	1.9	1.7	1.6	2.0	2.2	1.5	0.058	0.008	0.083
	Os08g23150	38.0	39.5	42.0	32.5	36.6	37.3	0.038	0.162	0.807
	Os08g34720	122.7	114.1	118.6	97.8	87.6	66.7	0.000	0.039	0.069
	Os08g37140	38.4	41.4	42.9	48.4	50.6	49.8	0.001	0.258	0.667
	Os08g41780	47.2	39.6	28.2	30.8	23.5	12.8	0.024	0.077	0.996
	Os08g42390	2.7	2.6	3.8	1.8	2.7	3.4	0.268	0.041	0.549
	Os09g01590	7.6	6.5	6.6	9.2	8.6	7.3	0.000	0.003	0.065
	Os09g11510	5.8	5.4	6.6	3.2	3.9	4.8	0.013	0.233	0.754
	Os09g25580	30.2	33.8	34.8	24.7	26.8	33.2	0.002	0.003	0.127
	Os09g39870	13.8	13.8	14.7	15.0	16.2	15.0	0.011	0.408	0.111
	Os10g27330	4.8	6.4	8.7	3.3	3.4	6.4	0.001	0.000	0.252
	Os10g41270	1.9	3.7	3.3	2.6	4.4	3.0	0.201	0.004	0.293
	Os11g04320	1.7	1.4	1.4	4.1	3.3	5.2	0.000	0.081	0.092
	Os11g12800	9.0	8.7	7.9	13.1	12.6	12.2	0.002	0.638	0.985
	Os11g45400	1.8	1.7	2.8	1.5	1.6	1.2	0.027	0.427	0.081
	Os12g01030	1.3	1.4	1.1	0.8	1.6	1.0	0.348	0.033	0.177
	Os12g04120	1.9	1.0	1.7	5.2	5.2	6.2	0.000	0.237	0.374
	Os12g38780	11.6	10.2	9.0	12.5	12.1	10.7	0.000	0.000	0.114
	Os01g02150	106.9	105.8	101.9	151.9	139.8	117.6	0.003	0.126	0.271
	Os01g02900	2.8	2.5	5.4	1.8	2.8	2.3	0.006	0.014	0.010
	Os01g02910	2.8	2.6	3.0	3.1	3.9	3.2	0.017	0.397	0.072
	Os01g02940	57.3	65.8	58.8	43.6	51.6	73.9	0.293	0.035	0.028
	Os01g03950	58.5	59.5	69.5	45.7	47.7	49.1	0.000	0.001	0.014
Glycosyl	Os01g04290	0.7	0.8	1.1	1.8	1.7	1.1	0.046	0.844	0.314
	Os01g05060	27.7	35.0	39.9	23.4	27.5	36.1	0.064	0.013	0.784
	Os01g07530	2.6	4.6	3.5	1.0	1.6	1.5	0.016	0.364	0.700
	Os01g08110	0.8	0.7	0.8	0.5	0.6	0.7	0.046	0.384	0.606
	Os01g10840	78.9	70.8	68.1	81.7	77.3	74.1	0.007	0.003	0.481
	Os01g16310	0.1	0.3	0.2	0.0	0.0	0.3	0.047	0.040	0.029
	Os01g17020	31.0	31.8	30.1	35.0	34.1	32.9	0.028	0.444	0.813
	Os01g19140	3.7	4.0	3.3	0.2	0.3	0.1	0.000	0.206	0.640
	Os01g33420	23.2	22.7	22.0	24.7	26.4	24.0	0.020	0.309	0.491
	Os01g43380	47.1	41.0	36.4	38.6	33.1	28.2	0.001	0.003	0.986
	Os01g46430	0.6	0.3	0.9	0.2	0.3	0.3	0.014	0.164	0.084
	Os01g51570	19.2	37.2	49.4	8.4	20.4	14.9	0.029	0.173	0.437
	Os01g52710	16.9	18.1	11.0	8.2	7.0	8.5	0.008	0.433	0.247
	Os01g56460	20.1	22.2	22.2	22.6	24.4	26.5	0.003	0.020	0.380
	Os01g56670	8.9	7.9	8.1	8.7	9.3	9.0	0.022	0.688	0.066
	Os01g64100	2.6	2.6	3.1	6.3	7.4	6.3	0.003	0.840	0.735
	Os01g68324	112.8	106.6	118.5	114.1	127.3	119.5	0.039	0.353	0.052
	Os01g69290	38.9	43.2	52.7	35.3	39.2	49.1	0.108	0.003	0.995
	Os01g71380	0.1	0.1	0.2	0.4	0.7	0.5	0.010	0.509	0.512
	Os01g71474	2.3	1.4	2.5	1.0	1.1	0.6	0.041	0.744	0.355
	Os01g71650	0.0	0.2	0.4	0.2	0.0	0.0	0.005	0.090	0.001
	Os01g71810	2.2	2.4	3.2	0.5	1.4	1.0	0.000	0.048	0.129
	Os01g71820	2.7	2.5	3.5	1.0	0.8	1.5	0.002	0.238	0.939
	Os01g71860	0.1	1.7	2.2	0.1	0.1	0.0	0.028	0.245	0.188
	Os02g03850	13.5	12.8	14.5	13.4	15.0	13.4	0.173	0.233	0.004
	Os02g04250	55.3	55.2	52.3	43.9	44.5	48.5	0.017	0.973	0.477
	Os02g07480	9.4	8.4	6.8	9.0	7.6	6.4	0.183	0.003	0.860
	Os02g11110	6.2	6.2	5.0	3.3	2.6	0.5	0.001	0.054	0.527
	Os02g17880	5.3	5.9	6.1	2.6	3.0	3.7	0.003	0.451	0.935
	Os02g22190	24.6	23.4	24.6	28.0	24.4	20.7	0.811	0.019	0.019

Glycosyl	Os02g29530	38.6	39.1	41.7	49.9	52.7	52.4	0.000	0.446	0.780
	Os02g32750	48.8	51.2	47.5	40.2	39.4	42.9	0.000	0.811	0.088
	Os02g33000	2.2	6.5	2.9	2.6	4.2	12.9	0.042	0.014	0.006
	Os02g38160	32.9	24.3	25.4	27.2	23.5	21.9	0.062	0.018	0.424
	Os02g41520	8.6	8.3	8.3	7.6	6.9	7.7	0.048	0.579	0.708
	Os02g46910	168.7	115.7	152.0	121.9	73.8	117.5	0.013	0.028	0.913
	Os02g50780	9.0	7.7	7.5	9.8	9.2	8.8	0.035	0.123	0.765
	Os02g51130	4.1	2.6	2.8	6.0	4.1	2.0	0.181	0.023	0.187
	Os02g51680	8.1	4.6	5.6	14.0	9.9	6.9	0.001	0.002	0.063
	Os02g56320	4.5	3.6	3.6	8.3	8.3	7.1	0.000	0.136	0.492
	Os03g02610	31.5	36.3	41.7	25.6	28.5	28.9	0.002	0.054	0.324
	Os03g08600	11.5	10.6	8.8	35.3	33.7	15.5	0.003	0.088	0.179
	Os03g10440	0.8	0.5	0.8	0.3	0.3	0.3	0.030	0.744	0.583
	Os03g10469	2.6	2.7	2.4	3.2	3.1	3.0	0.020	0.538	0.905
	Os03g11720	69.7	61.9	66.1	73.5	72.8	74.6	0.001	0.099	0.179
	Os03g12620	5.1	6.1	5.3	4.3	4.7	4.3	0.004	0.110	0.572
	Os03g13560	46.1	47.0	50.4	40.6	45.0	53.2	0.166	0.001	0.037
	Os03g13570	56.2	71.9	71.0	58.7	49.7	52.4	0.025	0.713	0.113
	Os03g15840	39.6	33.5	40.0	33.3	33.5	30.8	0.008	0.257	0.072
	Os03g19310	1.1	1.1	1.2	0.7	0.4	0.6	0.009	0.787	0.686
	Os03g21250	21.5	19.3	19.6	24.4	23.5	24.4	0.004	0.414	0.694
	Os03g25790	6.4	5.1	3.5	8.7	5.1	2.6	0.514	0.003	0.190
	Os03g26910	2.6	3.8	3.6	1.0	3.4	4.3	0.392	0.022	0.256
	Os03g37010	25.1	23.5	21.9	16.6	17.7	21.1	0.042	0.931	0.325
	Os03g59430	0.6	0.4	0.4	1.7	0.9	0.2	0.073	0.036	0.075
	Os03g60939	30.2	29.7	29.5	22.0	21.6	22.6	0.000	0.793	0.654
	Os04g01280	6.1	5.2	5.3	6.7	7.1	6.1	0.049	0.459	0.485
	Os04g12010	0.3	0.2	0.2	0.6	0.3	0.1	0.097	0.021	0.076
	Os04g30770	8.2	9.1	10.0	6.2	6.6	10.7	0.065	0.008	0.112
	Os04g32370	5.6	4.3	4.5	6.1	5.8	4.5	0.060	0.020	0.165
	Os04g33640	47.0	42.3	18.2	14.9	6.6	15.7	0.028	0.426	0.263
	Os04g40490	0.8	0.5	0.6	1.8	1.7	2.0	0.000	0.236	0.324
	Os04g40500	4.8	4.8	5.2	2.5	2.2	2.7	0.000	0.066	0.623
	Os04g40510	22.9	27.2	27.4	10.7	12.2	16.8	0.000	0.003	0.119
	Os04g40520	16.6	15.9	15.2	20.5	20.5	23.8	0.000	0.125	0.011
	Os04g42290	32.0	32.1	33.3	28.1	27.3	29.8	0.018	0.487	0.924
	Os04g43700	11.8	10.8	9.7	12.4	12.0	9.8	0.130	0.003	0.400
	Os04g44740	2.2	1.8	1.6	3.6	3.1	1.2	0.016	0.004	0.023
	Os04g49960	26.1	25.7	23.8	33.2	32.3	31.2	0.001	0.325	0.952
	Os04g51450	2.8	3.5	3.5	1.7	2.3	1.3	0.003	0.267	0.317
	Os04g51690	47.4	45.2	46.3	46.9	51.1	49.2	0.025	0.718	0.083
	Os04g53210	194.7	150.1	163.4	195.6	150.0	95.2	0.070	0.005	0.052
	Os04g57730	14.4	15.3	14.2	12.1	12.2	12.9	0.045	0.911	0.711
	Os05g03174	37.6	37.2	38.7	45.5	44.7	43.2	0.003	0.923	0.566
	Os05g10630	46.9	50.6	54.4	47.4	51.5	54.3	0.772	0.014	0.952
	Os05g11850	12.1	11.5	12.3	12.5	13.9	13.5	0.009	0.445	0.151
	Os05g15850	6.3	5.5	8.9	2.9	3.2	2.0	0.011	0.726	0.316
	Os05g23600	80.8	80.1	85.9	86.5	91.9	91.0	0.009	0.220	0.371
Glycosyl	Os05g23924	25.9	19.9	22.9	19.3	17.5	14.7	0.001	0.016	0.081
	Os05g25560	0.0	0.0	0.1	2.6	2.5	2.3	0.001	0.961	0.904
	Os05g27960	35.4	33.5	35.7	37.5	38.7	36.2	0.003	0.787	0.032
	Os05g35200	38.0	30.4	38.4	41.1	44.1	24.0	0.740	0.058	0.007
	Os05g37130	5.2	4.3	2.7	3.3	2.2	1.4	0.007	0.019	0.725
	Os05g40720	17.7	15.0	14.4	14.3	13.8	13.3	0.001	0.003	0.036
	Os05g48600	30.7	26.3	23.7	26.8	23.1	21.0	0.095	0.049	0.963
	Os06g04080	18.7	17.6	19.1	26.4	23.1	24.5	0.001	0.266	0.598

	Os06g07600	21.6	21.5	23.3	23.9	21.4	26.0	0.068	0.033	0.315
	Os06g22070	44.3	50.1	55.0	45.2	54.4	61.1	0.219	0.020	0.741
	Os06g25010	2.2	6.2	3.2	0.2	0.7	0.5	0.000	0.000	0.000
	Os06g27560	6.0	8.7	6.8	12.1	10.4	10.8	0.040	0.910	0.517
	Os06g34020	1.2	2.0	2.2	2.1	3.6	4.4	0.005	0.024	0.355
	Os06g36890	1.9	1.7	1.8	0.6	0.7	1.0	0.000	0.163	0.114
	Os06g40060	25.4	23.6	25.6	33.3	31.6	28.0	0.000	0.120	0.064
	Os06g43610	16.7	15.4	15.3	20.3	21.3	20.1	0.000	0.425	0.245
	Os06g44050	28.2	36.1	35.3	23.0	24.1	33.6	0.001	0.001	0.014
	Os06g46340	5.8	10.8	10.2	4.7	6.3	6.4	0.017	0.054	0.371
	Os06g48200	562.9	574.2	545.5	354.8	238.3	347.9	0.007	0.776	0.622
	Os06g49320	10.9	11.3	12.4	9.9	11.2	12.1	0.297	0.026	0.606
	Os06g51160	0.5	0.3	0.4	0.1	0.0	0.1	0.000	0.041	0.240
	Os07g01240	20.4	16.3	18.8	20.9	18.2	19.2	0.210	0.016	0.633
	Os07g05820	8.3	10.1	9.3	6.2	9.7	12.2	0.807	0.004	0.023
	Os07g10830	162.5	161.2	169.0	141.9	154.5	159.1	0.007	0.056	0.243
	Os07g10840	2.1	1.8	1.8	12.0	11.7	4.3	0.000	0.002	0.003
	Os07g23850	0.2	0.0	0.0	10.0	15.2	6.6	0.000	0.134	0.132
	Os07g27320	14.5	13.4	12.2	10.9	10.6	8.5	0.015	0.236	0.927
	Os07g28660	0.7	1.1	0.7	2.7	2.1	1.7	0.019	0.561	0.545
	Os07g34580	46.0	54.0	61.9	30.3	45.0	51.2	0.010	0.009	0.688
	Os07g43160	1.0	1.4	1.0	0.4	0.7	2.1	0.823	0.088	0.045
	Os07g46380	3.1	2.4	2.8	3.3	3.3	1.5	0.708	0.031	0.024
	Os07g48370	24.2	22.0	23.4	23.1	24.7	25.8	0.014	0.096	0.013
	Os07g48520	1.7	1.4	1.2	2.0	2.1	1.5	0.005	0.022	0.305
	Os07g49370	42.8	45.3	43.5	58.1	57.2	47.6	0.034	0.459	0.506
	Os08g21330	11.1	9.6	11.7	14.1	15.0	14.9	0.001	0.490	0.341
	Os08g23780	26.3	22.2	23.2	29.7	28.3	28.9	0.009	0.290	0.682
	Os08g40690	0.2	0.9	0.8	0.1	0.5	0.6	0.141	0.026	0.689
	Os09g08072	4.0	3.8	4.0	1.2	1.6	1.5	0.000	0.676	0.104
	Os09g12530	4.3	3.9	3.7	3.6	4.3	3.8	0.809	0.178	0.034
	Os09g25290	4.2	1.9	2.5	5.7	3.7	1.5	0.067	0.001	0.025
	Os09g28110	15.5	14.0	14.0	19.1	19.8	15.8	0.000	0.012	0.037
	Os09g29404	10.8	10.9	9.8	8.8	8.6	8.7	0.000	0.159	0.179
	Os09g30280	27.2	21.0	22.0	27.5	21.6	23.8	0.312	0.002	0.718
	Os09g33510	28.9	23.1	25.7	27.4	27.5	24.8	0.429	0.042	0.045
	Os09g34920	5.1	10.5	10.1	9.2	11.0	17.0	0.000	0.000	0.002
	Os09g36180	3.5	3.1	3.0	6.0	6.0	4.9	0.000	0.125	0.387
	Os09g36190	44.0	45.4	43.8	56.1	55.0	49.6	0.000	0.074	0.147
	Os09g36280	101.9	122.1	124.9	83.6	86.1	121.1	0.016	0.015	0.158
Glycosyl	Os09g38500	29.4	34.5	35.1	43.4	45.8	49.1	0.000	0.011	0.508
	Os10g02770	63.5	58.1	80.2	75.6	79.7	102.1	0.005	0.009	0.606
	Os10g07290	82.3	84.0	100.5	81.1	81.6	75.5	0.037	0.365	0.058
	Os10g13810	8.0	9.0	4.8	3.5	3.0	2.5	0.003	0.135	0.304
	Os10g31650	0.5	0.5	0.3	0.1	0.0	0.0	0.028	0.685	0.876
	Os10g39900	17.4	16.0	15.0	19.3	19.1	17.2	0.010	0.079	0.773
	Os10g40640	11.2	10.5	4.8	2.0	1.3	2.0	0.004	0.276	0.225
	Os10g41250	3.9	5.6	4.6	7.8	8.0	4.8	0.005	0.039	0.065
	Os11g03160	12.2	9.7	10.1	12.7	12.0	12.4	0.003	0.022	0.123
	Os11g04900	1.1	0.9	0.6	0.0	0.1	0.0	0.000	0.148	0.160
	Os11g27400	6.5	5.6	7.5	4.1	4.8	4.8	0.005	0.267	0.286
	Os11g33270	86.0	94.6	100.3	37.2	48.6	46.8	0.001	0.417	0.918
	Os11g44950	62.2	70.4	65.7	73.8	55.7	38.2	0.029	0.027	0.011
	Os11g47510	0.3	0.7	0.5	0.0	0.0	0.0	0.000	0.065	0.067
	Os11g47520	0.8	3.3	2.1	0.2	1.1	0.3	0.001	0.004	0.103
	Os12g02910	16.1	13.0	13.7	15.4	14.7	14.9	0.151	0.037	0.175

Os12g04210	15.9	17.1	18.1	15.7	18.1	21.0	0.240	0.046	0.412
Os12g10910	1.0	0.9	1.0	0.1	0.1	0.0	0.000	0.877	0.792
Os12g13640	4.4	3.7	3.7	8.2	6.8	4.4	0.000	0.005	0.021
Os12g29990	14.0	13.5	13.3	11.7	12.0	9.8	0.003	0.136	0.362
Os12g36870	0.9	0.6	0.8	1.3	1.9	2.3	0.002	0.312	0.158
Os12g38930	17.5	16.8	18.4	17.2	18.6	21.3	0.009	0.005	0.044
Os12g40550	46.7	49.0	49.9	86.8	102.4	94.1	0.000	0.087	0.198
