# Supplementary material for: Transcriptomic and Co-Expression Network Profiling of Shoot Apical Meristem Reveal Contrasting Response to Nitrogen Rate between Indica and Japonica Rice Subspecies
Source: Int J Mol Sci. 2019 Nov 25;20(23):5922. doi: 10.3390/ijms20235922 (PMC6928681; doi:10.3390/ijms20235922)
Supplement: Supplementary file 1 [file ijms-20-05922-s001.zip › Figure S1-12 + Table S1-15/Figure S12.pdf]

Figure S12. Different expression level (FPKM) of selected genes related to N metabolism and transport between variety and in response to N rate.

	Locus	NPB			YD6			*P* value		
	LOC_#	LN	MN	HN	LN	MN	HN	Variety	N rate	Variety x N
Ammonium	Os01g65000	2.5	2.1	1.2	1.6	1.2	0.6	0.027	0.028	0.856
	Os02g34580	0.4	0.5	0.4	0.2	0.3	0.2	0.007	0.268	0.708
	Os02g40730	4.5	4.3	3.6	6.1	14.9	10.0	0.013	0.214	0.200
	Os03g62200	1.8	2.3	2.5	0.4	0.7	0.4	0.001	0.497	0.669
	Os05g39240	0.7	0.9	0.5	0.3	0.3	0.4	0.011	0.453	0.421
	Os12g01420	0.7	0.7	0.8	0.2	0.2	0.2	0.004	0.961	0.905
Nitrate and nitrite	Os01g50820	3.3	1.8	1.5	8.6	5.8	4.7	0.001	0.049	0.522
	Os02g22020	2.6	4.8	7.5	3.4	4.4	6.5	0.734	0.002	0.419
	Os02g53130	2.7	2.8	1.5	7.4	4.2	1.1	0.020	0.007	0.034
	Os03g13274	20.4	15.3	13.7	27.2	25.8	15.9	0.004	0.007	0.145
	Os04g31030	7.2	6.0	6.9	3.7	3.2	2.9	0.000	0.323	0.507
	Os04g52950	3.0	2.0	2.1	3.6	4.3	3.6	0.009	0.633	0.269
	Os06g17870	68.2	64.2	50.4	224.2	194.6	152.3	0.000	0.099	0.355
	Os08g36480	0.3	0.5	0.4	0.8	0.8	1.4	0.010	0.317	0.330
	Os08g36500	0.1	0.2	0.2	0.3	0.5	0.5	0.009	0.392	0.553
	Os09g07920	48.6	48.4	51.4	68.9	59.2	53.6	0.010	0.275	0.124
	Os10g40600	6.7	6.3	6.9	13.4	14.3	13.2	0.000	0.819	0.265
	Os05g42350	59.9	83.5	111.1	56.1	82.7	103.6	0.589	0.004	0.928
Glutamate	Os06g08890	1.7	1.4	1.5	0.0	0.0	0.0	0.000	0.645	0.452
	Os06g08910	4.3	3.9	4.0	1.7	1.9	1.9	0.000	0.932	0.499
	Os06g09090	5.3	4.4	5.1	0.4	0.4	0.4	0.000	0.431	0.451
	Os06g09120	0.8	0.7	0.7	1.5	1.4	1.4	0.000	0.716	1.000
	Os06g09130	9.7	8.0	8.1	0.4	0.7	0.4	0.000	0.045	0.021
	Os06g46670	7.5	8.0	8.2	6.5	8.0	9.4	0.907	0.025	0.124
	Os07g01310	2.2	1.9	1.8	3.0	3.0	3.0	0.001	0.675	0.591
	Os07g27790	34.2	33.7	35.6	29.5	31.3	29.9	0.022	0.873	0.632
	Os07g33790	23.3	20.3	20.1	12.1	13.1	10.3	0.000	0.000	0.001
	Os07g46460	23.8	19.4	20.4	23.4	25.6	22.5	0.001	0.030	0.003
	Os09g25960	0.2	0.2	0.2	0.9	1.0	0.7	0.000	0.198	0.186
	Os09g25980	0.4	0.3	0.2	1.1	1.1	0.8	0.000	0.004	0.227
Asparagine	Os01g10960	5.0	5.7	4.3	3.7	3.8	3.2	0.008	0.168	0.685
	Os01g11010	14.6	15.4	14.4	45.4	41.4	29.4	0.000	0.041	0.050
	Os03g18130	4.1	5.2	4.6	5.4	5.9	6.6	0.024	0.322	0.539
	Os05g32820	14.1	12.0	10.9	14.0	8.9	5.4	0.006	0.001	0.054
	Os06g15420	89.9	80.3	87.6	73.4	75.9	84.7	0.000	0.003	0.004
	Os06g41390	13.7	13.6	13.4	14.4	15.3	14.7	0.029	0.679	0.664
	Os07g31460	17.5	15.8	15.5	18.7	18.0	16.8	0.003	0.008	0.487
Glutamine	Os02g08270	25.5	44.3	23.4	0.8	2.9	5.4	0.000	0.077	0.063
	Os02g47850	74.4	77.1	87.8	56.5	64.4	62.1	0.000	0.003	0.019
	Os02g50240	254.5	266.4	274.5	141.8	186.6	225.7	0.000	0.014	0.096
	Os03g48060	14.4	22.1	27.0	8.0	15.8	31.0	0.307	0.004	0.252
	Os03g50490	2.1	2.9	1.9	1.3	1.8	2.3	0.019	0.044	0.019
	Os04g56400	8.9	11.2	14.8	6.1	9.2	14.2	0.136	0.004	0.698
	Os07g07260	22.4	21.7	22.7	20.7	23.6	21.1	0.254	0.100	0.009
	Os08g23730	67.9	73.6	83.6	66.6	74.2	81.1	0.551	0.001	0.779
Tryptoph	Os01g07500	24.3	24.0	27.1	19.5	23.4	22.1	0.032	0.265	0.351
	Os02g16630	3.2	3.6	3.9	3.5	4.6	6.3	0.005	0.008	0.072
	Os04g52479	74.3	57.6	57.6	137.3	148.2	160.7	0.000	0.315	0.004
	Os06g42560	7.0	12.8	13.2	3.2	7.0	8.9	0.000	0.000	0.397
	Os07g12330	9.4	10.8	11.8	3.4	5.1	4.9	0.000	0.061	0.624

an	Os08g04180	43.1	50.7	43.7	25.5	35.6	46.1	0.005	0.022		0.026	
	Os08g04540	0.6	5.0	2.4	0.9	1.6	4.5	0.661	0.026		0.038	
	Os08g04560	2.7	5.5	5.5	2.4	3.2	5.9	0.055	0.001		0.034	
Methionine	Os01g18860	1.8	0.4	0.8	34.5	33.1	26.3	0.000		0.111		0.159
	Os01g22010	373.2	503.2	520.4	325.1	484.8	680.3	0.437	0.004			0.123
	Os02g39795	2.3	3.4	2.5	3.1	1.2	1.6	0.066		0.398	0.033	
	Os02g57990	26.7	26.0	24.8	31.5	31.4	24.8	0.000	0.000		0.003	
	Os03g04370	15.1	16.5	13.3	13.3	10.1	9.8	0.004		0.113		0.177
	Os03g24600	48.0	59.1	57.3	53.3	60.7	68.2	0.037	0.010			0.300
	Os04g42095	143.4	141.9	145.0	237.1	241.0	296.7	0.000		0.312		0.360
	Os05g01470	11.4	10.8	14.7	9.7	11.8	12.8	0.240	0.024			0.257
	Os05g04510	398.2	425.9	444.2	397.2	447.9	483.4	0.276	0.048			0.638
	Os05g04990	2.1	2.1	1.7	3.6	2.7	1.7	0.022	0.015			0.105
	Os05g33510	27.7	34.1	33.3	28.3	31.2	35.1	0.894	0.018			0.350
	Os06g04650	34.7	34.6	31.1	37.6	33.9	27.3	0.648	0.006			0.125
	Os09g25625	16.2	14.4	15.9	33.5	36.7	49.1	0.004		0.483		0.509
Asp	Os02g33780	25.4	24.7	24.2	9.8	8.2	7.4	0.000		0.248		0.805
	Os08g15030	32.0	36.0	43.5	31.1	33.6	41.0	0.364	0.010			0.931
NADH	Os01g07910	79.8	85.5	87.5	67.4	71.7	58.7	0.000		0.244		0.076
	Os01g59930	62.2	70.9	76.4	58.6	77.9	94.6	0.041	0.001		0.050	
	Os01g61410	9.5	10.2	10.5	5.5	6.6	7.1	0.000	0.040			0.739
	Os03g09210	93.2	112.4	114.9	114.2	115.9	117.2	0.017	0.020			0.054
	Os03g19890	150.6	175.2	171.1	231.1	240.5	240.7	0.000	0.015			0.277
	Os05g26660	1.5	1.4	1.6	0.1	0.1	0.1	0.000		0.647		0.644
	Os05g40990	14.4	15.0	17.4	12.6	13.1	12.5	0.000		0.072	0.041	
	Os05g43360	140.5	156.9	170.9	176.3	188.7	195.4	0.000	0.002			0.414
	Os05g48200	0.3	0.3	0.3	0.5	0.5	0.5	0.008		0.986		0.883
	Os06g47000	5.3	4.5	5.1	6.1	6.2	5.8	0.010		0.594		0.292
	Os07g37730	4.5	10.4	4.2	0.9	1.2	6.9	0.050		0.202		0.034
	Os07g45090	121.0	127.9	142.5	120.0	133.0	137.2	0.931	0.044			0.685
	Os08g04630	9.3	11.8	9.5	7.5	7.7	11.5	0.037	0.033		0.006	
	Os08g06430	125.4	151.0	152.9	136.5	151.1	164.0	0.178	0.009			0.592
	Os08g23810	110.3	118.3	134.9	92.0	117.1	132.0	0.063	0.001			0.142
	Os09g10600	37.3	42.0	50.7	38.4	43.9	55.1	0.388	0.010			0.872
	Os10g42840	192.4	213.5	217.4	178.1	193.0	190.3	0.004	0.024			0.541
	Os12g33958	1.0	2.0	2.2	0.8	1.0	1.9	0.139		0.045		0.525
	Os12g34014	1.2	2.9	4.2	1.4	1.5	2.8	0.078	0.009			0.236
	Os12g34094	0.8	1.4	1.2	1.0	1.5	2.7	0.011	0.004		0.017	
Proline	Os01g07520	21.4	18.9	19.6	21.4	22.2	21.1	0.002	0.072		0.014	
	Os01g57004	43.0	34.4	39.3	40.0	31.1	29.0	0.043	0.033			0.356
	Os01g72205	2.5	2.4	3.1	4.0	3.8	4.6	0.007		0.330		0.977
	Os02g16640	49.8	50.3	51.1	52.0	57.9	68.3	0.003	0.024		0.046	
	Os02g30640	13.7	14.1	14.3	19.7	20.7	21.7	0.000		0.516		0.808
Proline	Os03g13560	46.1	47.0	50.4	40.6	45.0	53.2	0.166	0.001		0.037	
	Os03g24940	43.0	36.1	36.0	44.5	35.1	35.8	0.906	0.002			0.708
	Os03g29920	48.2	36.1	32.3	61.2	36.5	24.4	0.683	0.007			0.226
	Os03g31570	18.7	17.1	18.1	36.9	33.5	33.0	0.000		0.269		0.569
	Os04g32370	5.6	4.3	4.5	6.1	5.8	4.5	0.060	0.020			0.165
	Os04g52504	18.8	28.8	20.3	18.3	12.1	6.7	0.038		0.375		0.269
	Os05g05740	34.6	43.1	47.3	33.4	41.4	38.2	0.050	0.008			0.170
	Os05g46830	0.3	0.7	0.5	0.0	0.0	0.0	0.001		0.324		0.324
	Os05g46840	0.8	1.6	0.9	0.2	0.1	0.5	0.030		0.614		0.325
	Os06g21890	0.5	0.7	0.5	0.0	0.0	0.0	0.000		0.576		0.447
	Os07g23640	0.2	0.7	2.2	0.2	0.5	1.0	0.049	0.003			0.078
	Os07g23660	0.0	0.2	0.3	0.0	0.1	0.3	0.679	0.000			0.750
	Os07g41600	74.6	88.5	78.9	53.9	60.7	71.8	0.011		0.224		0.322

	Os07g48390	2.4	2.6	4.0	1.2	2.4	1.5	0.008	0.128		0.083
	Os07g48520	1.7	1.4	1.2	2.0	2.1	1.5	0.005	0.022		0.305
	Os09g23370	17.6	19.2	17.6	18.7	23.6	22.5	0.044	0.227		0.514
	Os09g28110	15.5	14.0	14.0	19.1	19.8	15.8	0.000	0.012	0.037	
	Os09g33510	28.9	23.1	25.7	27.4	27.5	24.8	0.429	0.042	0.045	
	Os10g05980	2.0	6.2	3.8	1.1	0.3	2.3	0.000	0.006	0.001	
	Os10g06000	2.9	8.7	5.7	2.9	2.2	3.2	0.002	0.032	0.012	
	Os10g40360	1.4	1.9	1.1	0.7	0.7	2.8	0.974	0.186	0.035	
Glycine	Os01g51410	55.8	44.3	49.4	28.4	31.2	38.4	0.001	0.235		0.089
	Os06g45670	45.9	53.8	57.3	54.9	61.3	65.0	0.002	0.003		0.909
	Os10g37180	21.6	29.8	41.4	17.1	29.9	54.8	0.564	0.008		0.361
Cysteine	Os03g11660	14.8	15.3	14.8	16.6	15.9	17.6	0.020	0.660		0.321
	Os03g12110	4.7	6.2	9.5	4.5	6.7	12.4	0.261	0.002		0.368
	Os06g05690	0.8	0.9	0.9	2.1	2.2	2.0	0.000	0.678		0.941
	Os06g05700	4.9	4.1	4.1	7.3	7.1	6.5	0.002	0.479		0.852
	Os06g36830	2.0	1.5	1.3	1.2	1.2	1.2	0.008	0.047		0.062
	Os06g36840	8.2	7.8	8.2	6.5	6.7	7.6	0.031	0.421		0.555
	Os06g36880	0.6	1.5	0.9	0.8	1.7	2.6	0.016	0.018		0.053
	Os12g42876	237.1	220.6	295.6	170.5	194.2	185.8	0.016	0.335		0.320
	Os12g42884	357.9	356.8	484.9	314.8	359.8	323.3	0.018	0.090	0.043	
	Os12g42980	57.3	86.7	115.2	22.5	43.7	61.8	0.000	0.002		0.508
