# Supplementary material for: Transcriptomic and Co-Expression Network Profiling of Shoot Apical Meristem Reveal Contrasting Response to Nitrogen Rate between Indica and Japonica Rice Subspecies
Source: Int J Mol Sci. 2019 Nov 25;20(23):5922. doi: 10.3390/ijms20235922 (PMC6928681; doi:10.3390/ijms20235922)
Supplement: Supplementary file 1 [file ijms-20-05922-s001.zip › Figure S1-12 + Table S1-15/Figure S2.pdf]

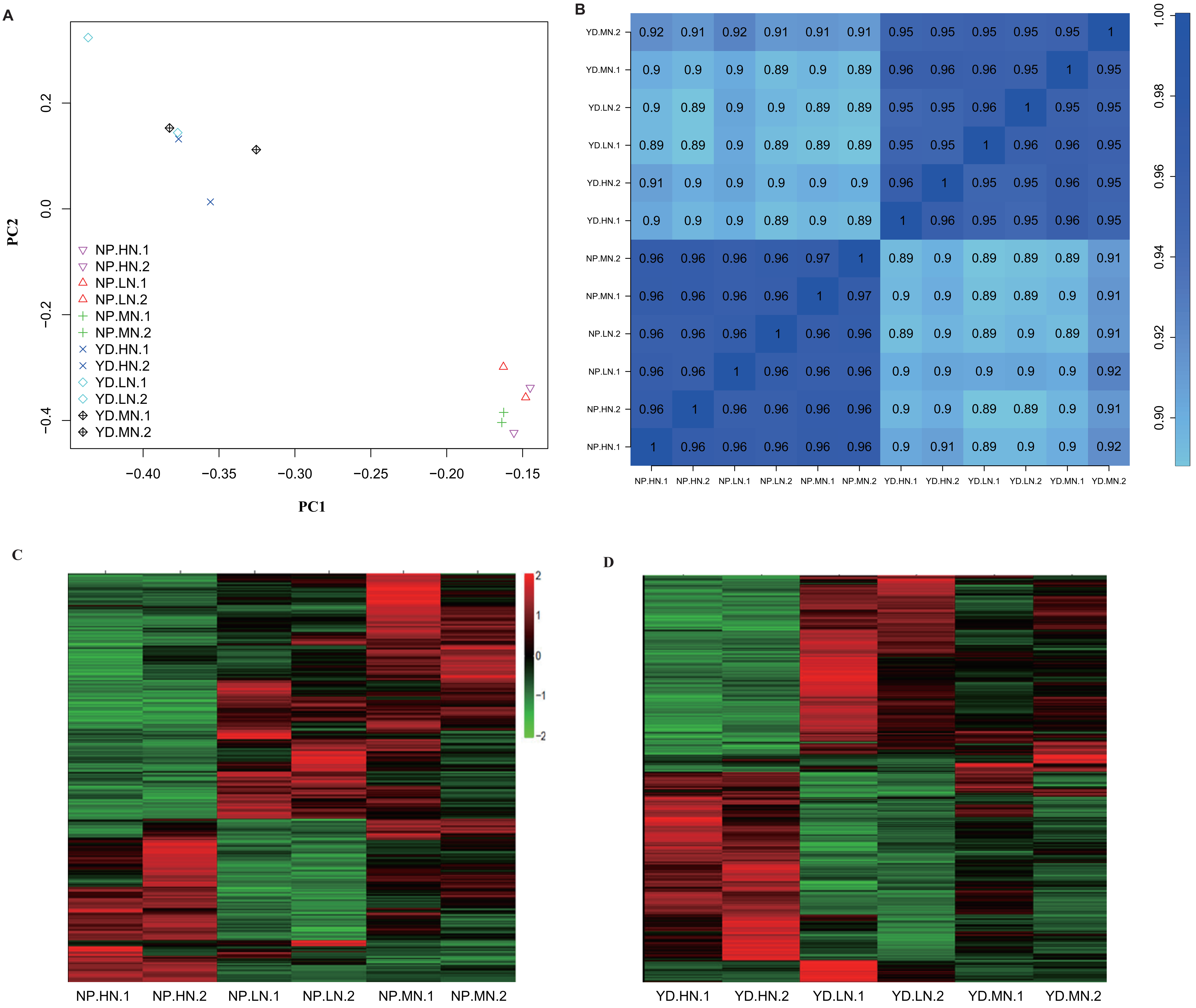

Figure S2. Global profiling of RNA sequencing results. (A) Principal Component Analysis (PCA) of the RNA-Seq data; (B) Cluster analysis of the samples; (C) Heatmap of RNA_Seq samples in NPB; (D) Heatmap of RNA_Seq samples in YD6.
