# Supplementary material for: Transcriptomic and Co-Expression Network Profiling of Shoot Apical Meristem Reveal Contrasting Response to Nitrogen Rate between Indica and Japonica Rice Subspecies
Source: Int J Mol Sci. 2019 Nov 25;20(23):5922. doi: 10.3390/ijms20235922 (PMC6928681; doi:10.3390/ijms20235922)
Supplement: Supplementary file 1 [file ijms-20-05922-s001.zip › Figure S1-12 + Table S1-15/Figure S3.pdf]

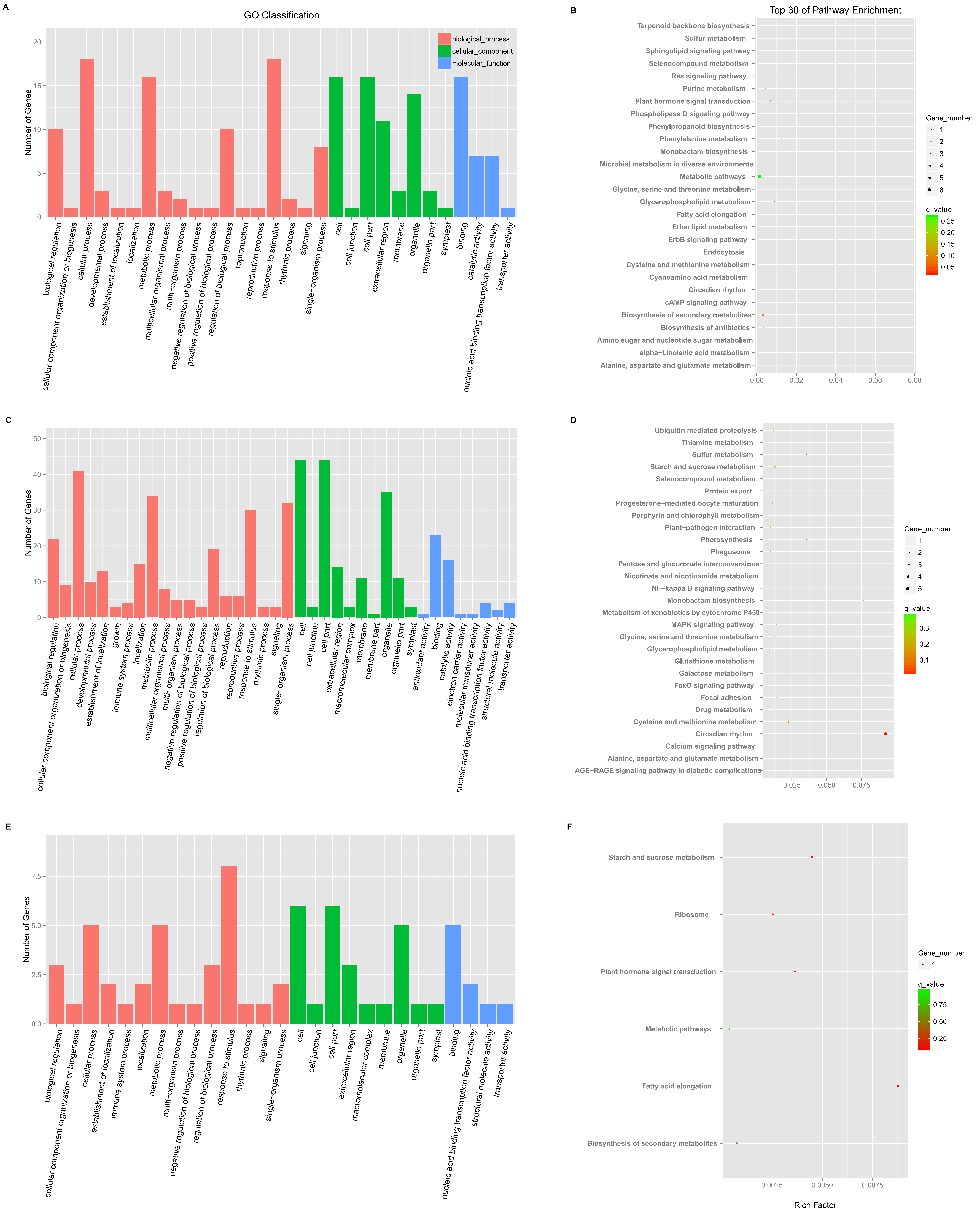

Figure S3. Functional analysis of differentially expressed genes (DEGs) under different N rate between YD6 and NPB. GO enrichment analysis and KEGG pathway of DEGs between varieties in LN (A, B), MN (C, D); and HN (E, F).
