# Supplementary material for: Transcriptomic and Co-Expression Network Profiling of Shoot Apical Meristem Reveal Contrasting Response to Nitrogen Rate between Indica and Japonica Rice Subspecies
Source: Int J Mol Sci. 2019 Nov 25;20(23):5922. doi: 10.3390/ijms20235922 (PMC6928681; doi:10.3390/ijms20235922)
Supplement: Supplementary file 1 [file ijms-20-05922-s001.zip › Figure S1-12 + Table S1-15/Figure S5.pdf]

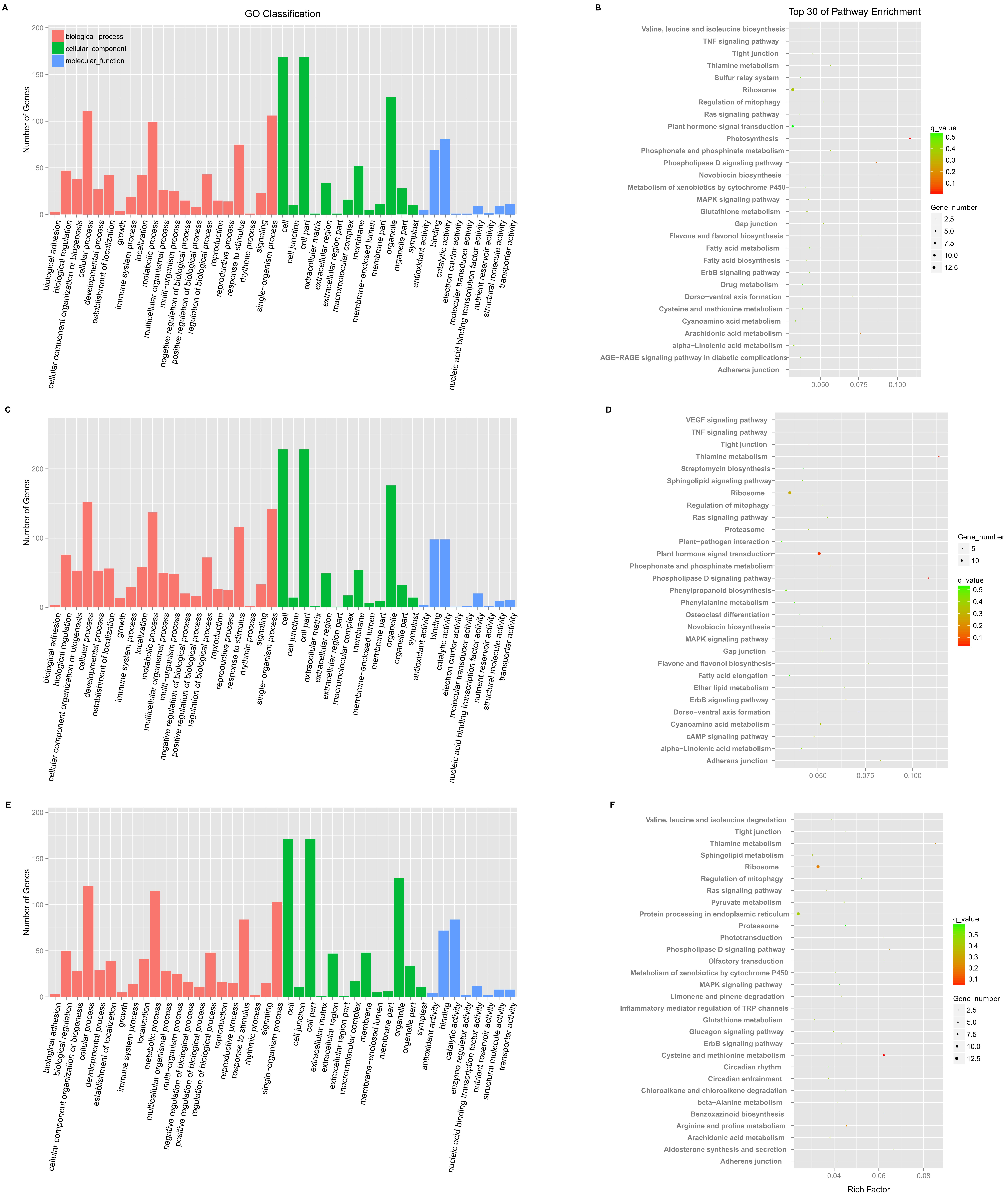

Figure S5. Functional analysis of differentially expressed genes (DEGs) in response to N rate in *japonica* rice NPB. GO enrichment analysis and KEGG pathway of DEGs in LN vs MN (A, B), L[illegible] vs HN (C, D); and MN vs HN (E, F).
