# Supplementary material for: Transcriptomic and Co-Expression Network Profiling of Shoot Apical Meristem Reveal Contrasting Response to Nitrogen Rate between Indica and Japonica Rice Subspecies
Source: Int J Mol Sci. 2019 Nov 25;20(23):5922. doi: 10.3390/ijms20235922 (PMC6928681; doi:10.3390/ijms20235922)
Supplement: Supplementary file 1 [file ijms-20-05922-s001.zip › Figure S1-12 + Table S1-15/Figure S6.pdf]

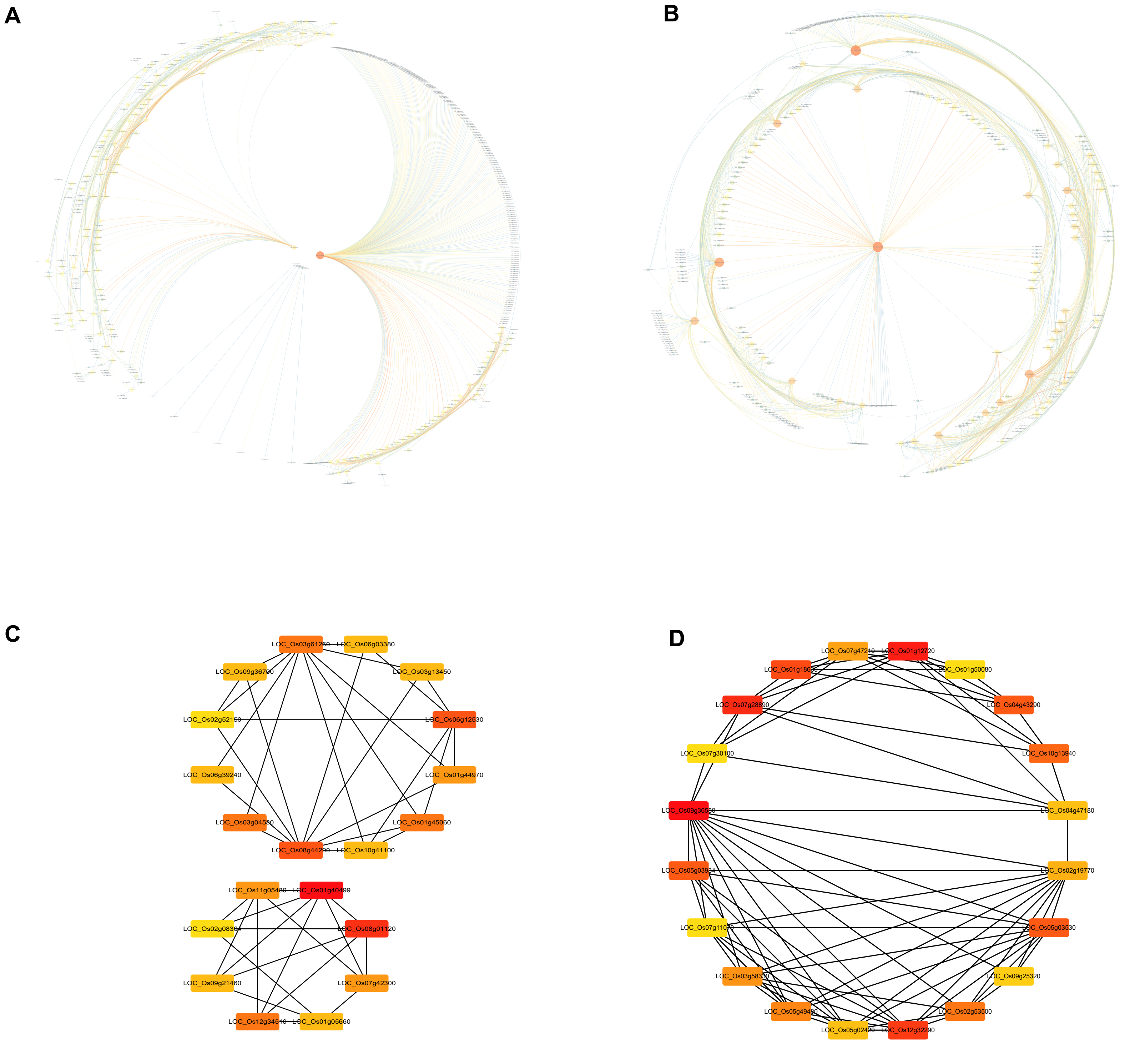

Figure S6. Co-expression network analysis of N content in leaf and stem related modules. (A, B) Gene co-expression networks of positive correlation blue module (A) and negative correlation green module (B) visualized using Cytoscape software platform. The circle size of and color depth indicate the degree of connectivity; (C, D) The correlation networks of top 20 nodes in blue module (C) and green module (D). The color depth represents the number of associated nodes.
