# Supplementary material for: Transcriptomic and Co-Expression Network Profiling of Shoot Apical Meristem Reveal Contrasting Response to Nitrogen Rate between Indica and Japonica Rice Subspecies
Source: Int J Mol Sci. 2019 Nov 25;20(23):5922. doi: 10.3390/ijms20235922 (PMC6928681; doi:10.3390/ijms20235922)
Supplement: Supplementary file 1 [file ijms-20-05922-s001.zip › Figure S1-12 + Table S1-15/Figure S7.pdf]

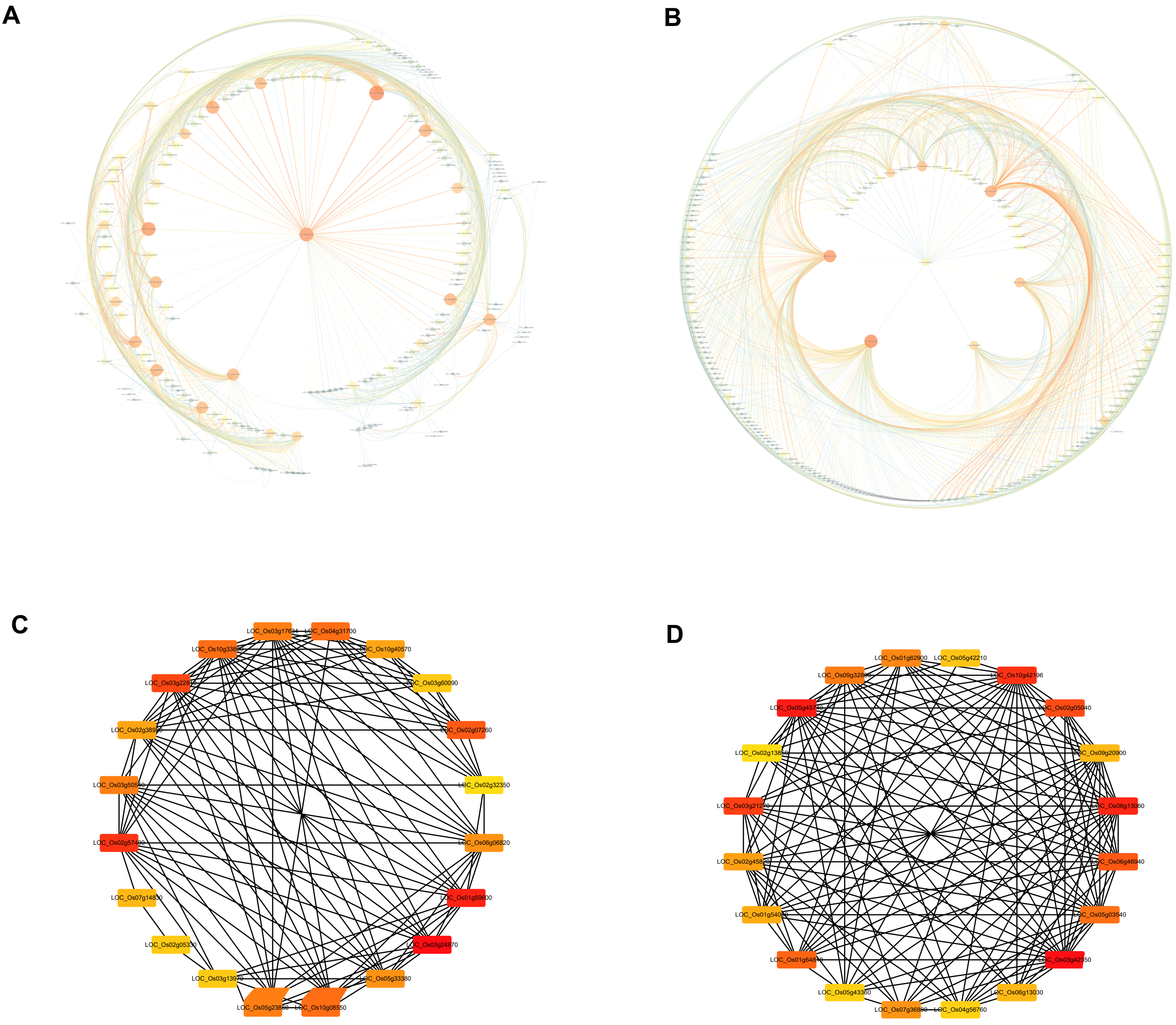

Figure S7. Co-expression network analysis of dry weight in root related modules. (A, B) Gene co-expression networks of positive correlation tan module (A) and negative correlation red module (B) visualized using Cytoscape software platform. T The circle size of and color depth indicate the degree of connectivity; (C, D) The correlation networks of top 20 nodes in blue tan (C) and red module (D). The color depth represents the number of associated nodes.
