# Supplementary material for: Transcriptomic and Co-Expression Network Profiling of Shoot Apical Meristem Reveal Contrasting Response to Nitrogen Rate between Indica and Japonica Rice Subspecies
Source: Int J Mol Sci. 2019 Nov 25;20(23):5922. doi: 10.3390/ijms20235922 (PMC6928681; doi:10.3390/ijms20235922)
Supplement: Supplementary file 1 [file ijms-20-05922-s001.zip › Figure S1-12 + Table S1-15/Figure S8.pdf]

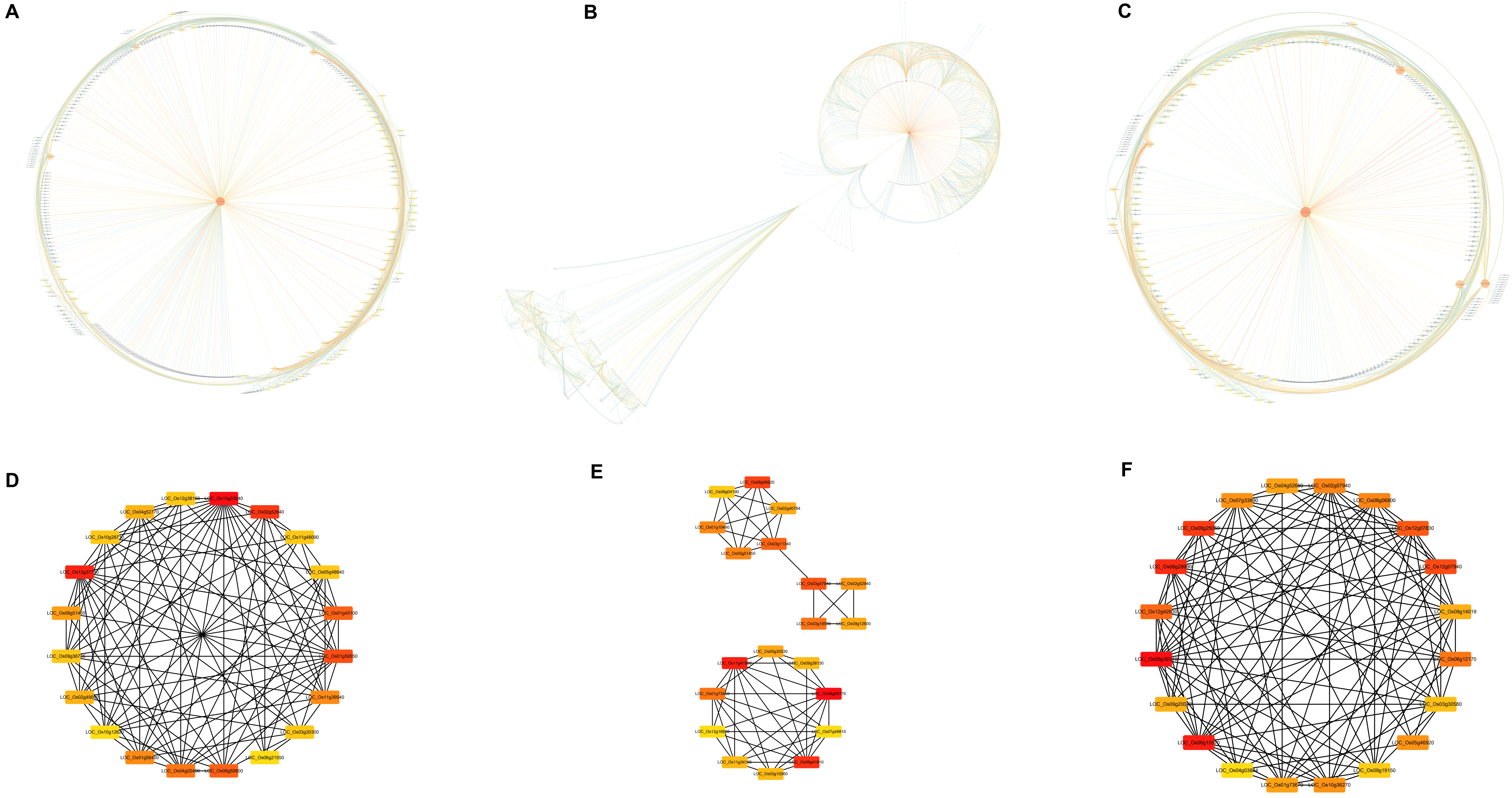

Figure S8. Co-expression network analysis of dry weight in stem and in leaf related modules. (A - C) Gene co-expression networks of positive correlation brown module (A), negative correlation pink module (B), and turquoise module (C) visualized using Cytoscape software platform. The circle size of and color depth indicate the degree of connectivity; (D - F) The correlation networks of top 20 nodes in brown module (D), pink module (E), and turquoise module (F). The color depth represents the number of associated nodes.
