# Supplementary material for: Transcriptomic and Co-Expression Network Profiling of Shoot Apical Meristem Reveal Contrasting Response to Nitrogen Rate between Indica and Japonica Rice Subspecies
Source: Int J Mol Sci. 2019 Nov 25;20(23):5922. doi: 10.3390/ijms20235922 (PMC6928681; doi:10.3390/ijms20235922)
Supplement: Supplementary file 1 [file ijms-20-05922-s001.zip › Figure S1-12 + Table S1-15/Figure S9.pdf]

Figure S9. Changes in the expression levels of hub genes in top ten node genes in blue, green, tan, red, brown, pink, and turquoise module, respectively.

Module	Hubgenes	Expression change between varieties			Expression change in response to N rate					
		YD6 vs NPB			YD6			NPB		
		LN	MN	HN	LN vs MN	LN vs HN	MN vs HN	LN vs MN	LN vs HN	MN vs HN
Blue	LOC_Os09g36700	0.14	0.07	0.33	0.27	-0.70	-0.41	0.37	-0.52	-0.13
	LOC_Os02g52150	0.05	0.15	0.52	0.43	-1.11	-0.68	0.35	-0.66	-0.29
	LOC_Os03g61260	-0.09	0.63	0.63	0.97	-2.25	-1.27	0.28	-1.54	-1.25
	LOC_Os11g34880	-1.03	0.24	0.81	3.23	-4.76	-1.52	1.99	-2.93	-0.92
	LOC_Os11g05480	-0.21	0.17	1.19	2.04	-4.41	-2.35	1.70	-3.02	-1.31
	LOC_Os03g07960	-1.02	0.25	1.07	1.67	-3.17	-1.49	0.43	-1.09	-0.65
	LOC_Os08g44290	-0.22	-0.07	1.73	1.28	-3.21	-1.91	1.17	-1.27	-0.09
	LOC_Os01g40499	-0.48	-0.40	-1.02	-0.21	0.84	0.64	-0.26	0.29	0.04
	LOC_Os10g41100	-0.62	-0.44	-0.82	-0.15	0.65	0.52	-0.30	0.45	0.16
	LOC_Os03g04530	-0.97	-1.38	-3.17	-0.70	2.81	2.12	-0.25	0.60	0.36
Green	LOC_Os05g03530	0.41	0.20	0.03	-0.62	1.04	0.44	-0.38	0.66	0.30
	LOC_Os10g13940	0.34	0.48	-0.29	-0.49	1.29	0.81	-0.60	0.65	0.07
	LOC_Os02g19770	0.34	0.28	-0.15	-0.47	1.13	0.68	-0.38	0.63	0.27
	LOC_Os02g53500	0.20	0.26	-0.43	-0.60	1.57	0.98	-0.63	0.93	0.31
	LOC_Os09g36580	0.42	0.28	-1.27	-0.87	2.78	1.92	-0.70	1.08	0.39
	LOC_Os07g28890	0.98	0.52	0.27	-0.94	1.82	0.90	-0.45	1.11	0.67
	LOC_Os01g18630	0.55	0.97	-0.04	-0.61	1.80	1.20	-1.01	1.20	0.20
	LOC_Os07g47210	0.87	0.74	0.29	-0.66	1.48	0.84	-0.49	0.89	0.41
	LOC_Os04g07280	0.78	1.05	-0.19	-0.49	1.74	1.26	-0.72	0.77	0.06
	LOC_Os01g12720	-0.31	-0.13	-0.05	0.30	-0.53	-0.22	0.14	-0.28	-0.12
Tan	LOC_Os01g59600	-0.02	0.07	-0.06	0.27	-0.36	-0.08	0.21	-0.40	-0.18
	LOC_Os03g22810	0.18	0.19	0.02	0.20	-0.30	-0.09	0.22	-0.47	-0.24
	LOC_Os02g07260	-0.03	0.02	-0.02	0.27	-0.36	-0.07	0.24	-0.35	-0.10
	LOC_Os03g50540	0.09	0.13	0.00	0.13	-0.20	-0.05	0.12	-0.30	-0.16
	LOC_Os02g57400	0.06	0.08	0.03	0.10	-0.19	-0.08	0.11	-0.23	-0.11
	LOC_Os05g23860	-0.11	-0.03	-0.18	0.32	-0.47	-0.13	0.28	-0.55	-0.26
	LOC_Os03g24870	-0.03	0.20	-0.04	0.23	-0.21	0.04	0.03	-0.23	-0.18
	LOC_Os04g31700	-0.09	0.04	-0.03	0.25	-0.39	-0.12	0.16	-0.34	-0.17
	LOC_Os10g33800	-0.09	-0.08	-0.06	-0.15	0.31	0.17	-0.13	0.33	0.21
	LOC_Os10g08550	-0.39	-0.54	-0.40	-0.85	1.72	0.89	-0.67	1.71	1.05
	LOC_Os01g64810	-0.86	-0.59	-0.55	-0.09	0.30	0.22	-0.33	0.60	0.28
	LOC_Os09g32800	-0.83	-0.97	-1.38	-0.49	0.95	0.47	-0.32	0.39	0.08

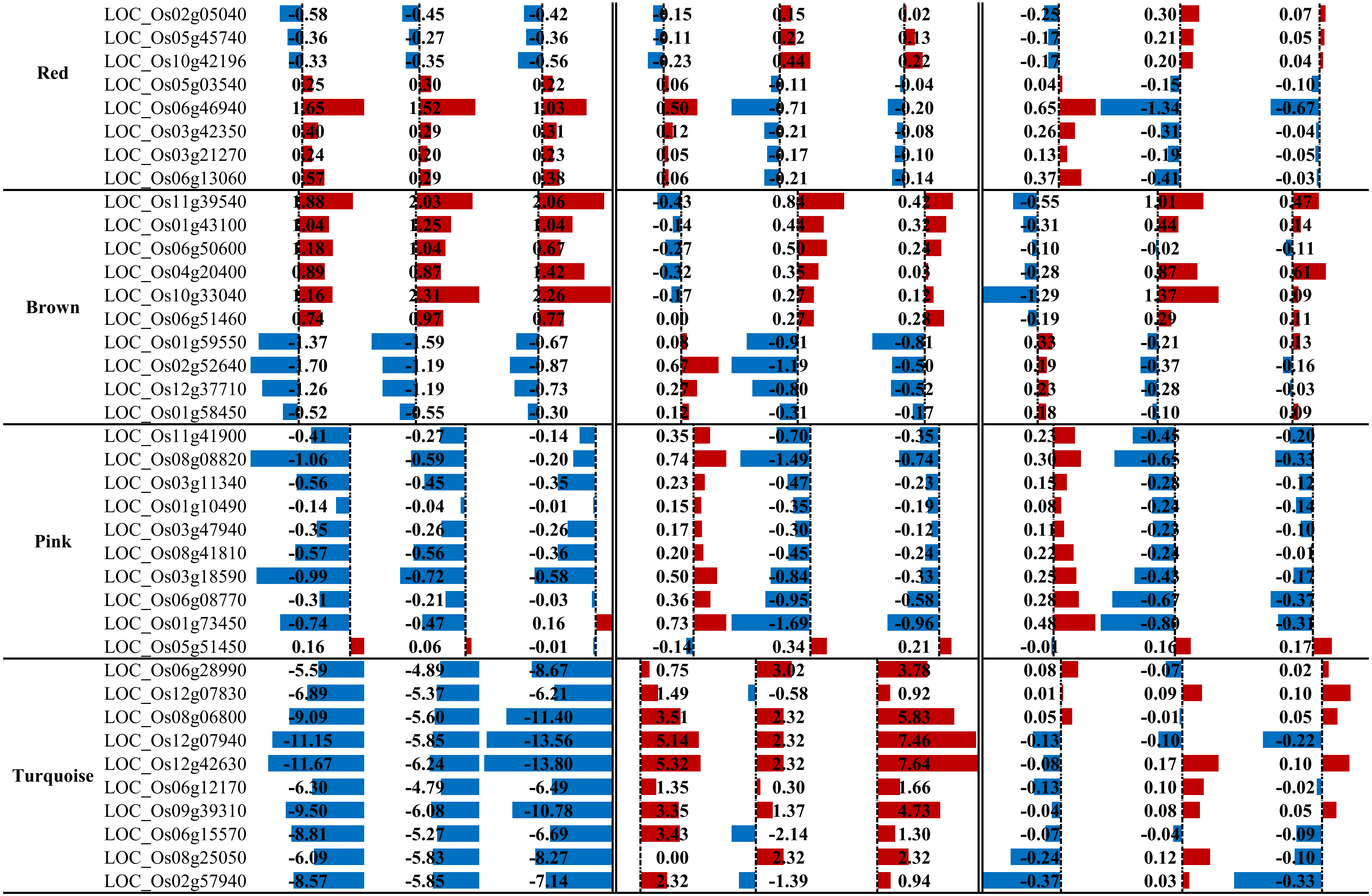
Red
LOC_Os02g05040 -0.58 -0.45 -0.42 -0.15 0.15 0.02 -0.25 0.30 0.07
LOC_Os05g45740 -0.36 -0.27 -0.36 -0.11 0.22 0.13 -0.17 0.21 0.05
LOC_Os10g42196 -0.33 -0.35 -0.56 -0.23 0.44 0.22 -0.17 0.20 0.04
LOC_Os05g03540 0.25 0.30 0.22 0.06 -0.11 -0.04 0.04 -0.15 -0.10
LOC_Os06g46940 1.65 1.52 1.03 0.50 -0.71 -0.20 0.65 -1.34 -0.67
LOC_Os03g42350 0.40 0.29 0.31 0.12 -0.21 -0.08 0.26 -0.31 -0.04
LOC_Os03g21270 0.24 0.20 0.23 0.05 -0.17 -0.10 0.13 -0.19 -0.05
LOC_Os06g13060 0.57 0.29 0.38 0.06 -0.21 -0.14 0.37 -0.41 -0.03
Brown
LOC_Os11g39540 1.88 2.03 2.06 -0.43 0.84 0.42 -0.55 1.01 0.47
LOC_Os01g43100 1.04 1.25 1.04 -0.14 0.44 0.32 -0.31 0.44 0.14
LOC_Os06g50600 1.18 1.04 0.67 -0.27 0.50 0.24 -0.10 -0.02 -0.11
LOC_Os04g20400 0.89 0.87 1.42 -0.32 0.35 0.03 -0.28 0.87 0.61
LOC_Os10g33040 1.16 2.31 2.26 -0.17 0.27 0.12 -1.29 1.37 0.09
LOC_Os06g51460 0.74 0.97 0.77 0.00 0.27 0.28 -0.19 0.29 0.11
LOC_Os01g59550 -1.37 -1.59 -0.67 0.08 -0.91 -0.81 0.33 -0.21 0.13
LOC_Os02g52640 -1.70 -1.19 -0.87 0.67 -1.19 -0.50 0.19 -0.37 -0.16
LOC_Os12g37710 -1.26 -1.19 -0.73 0.27 -0.80 -0.52 0.23 -0.28 -0.03
LOC_Os01g58450 -0.52 -0.55 -0.30 0.12 -0.31 -0.17 0.18 -0.10 0.09
Pink
LOC_Os11g41900 -0.41 -0.27 -0.14 0.35 -0.70 -0.35 0.23 -0.45 -0.20
LOC_Os08g08820 -1.06 -0.59 -0.20 0.74 -1.49 -0.74 0.30 -0.65 -0.33
LOC_Os03g11340 -0.56 -0.45 -0.35 0.23 -0.47 -0.23 0.15 -0.28 -0.12
LOC_Os01g10490 -0.14 -0.04 -0.01 0.15 -0.35 -0.19 0.08 -0.24 -0.14
LOC_Os03g47940 -0.35 -0.26 -0.26 0.17 -0.30 -0.12 0.11 -0.23 -0.10
LOC_Os08g41810 -0.57 -0.56 -0.36 0.20 -0.45 -0.24 0.22 -0.24 -0.01
LOC_Os03g18590 -0.99 -0.72 -0.58 0.50 -0.84 -0.33 0.25 -0.43 -0.17
LOC_Os06g08770 -0.31 -0.21 -0.03 0.36 -0.95 -0.58 0.28 -0.67 -0.37
LOC_Os01g73450 -0.74 -0.47 0.16 0.73 -1.69 -0.96 0.48 -0.80 -0.31
LOC_Os05g51450 0.16 0.06 -0.01 -0.14 0.34 0.21 -0.01 0.16 0.17
Turquoise
LOC_Os06g28990 -5.59 -4.89 -8.67 0.75 3.02 3.78 0.08 -0.07 0.02
LOC_Os12g07830 -6.89 -5.37 -6.21 1.49 -0.58 0.92 0.01 0.09 0.10
LOC_Os08g06800 -9.09 -5.60 -11.40 3.51 2.32 5.83 0.05 -0.01 0.05
LOC_Os12g07940 -11.15 -5.85 -13.56 5.14 2.32 7.46 -0.13 -0.10 -0.22
LOC_Os12g42630 -11.67 -6.24 -13.80 5.32 2.32 7.64 -0.08 0.17 0.10
LOC_Os06g12170 -6.30 -4.79 -6.49 1.35 0.30 1.66 -0.13 0.10 -0.02
LOC_Os09g39310 -9.50 -6.08 -10.78 3.35 1.37 4.73 -0.04 0.08 0.05
LOC_Os06g15570 -8.81 -5.27 -6.69 3.43 -2.14 1.30 -0.07 -0.04 -0.09
LOC_Os08g25050 -6.09 -5.83 -8.27 0.00 2.32 2.32 -0.24 0.12 -0.10
LOC_Os02g57940 -8.57 -5.85 -7.14 2.32 -1.39 0.94 -0.37 0.03 -0.33
